# Can intravitreal injections with higher volume cause higher intraocular eye pressure? Considerations for anti-complement injections in normal and glaucomatous eyes

**DOI:** 10.1186/s40942-023-00517-1

**Published:** 2023-12-14

**Authors:** Carsten H. Meyer, Gustavo Barreto Melo, Arshad M. Khanani

**Affiliations:** 1 Department of Ophthalmology, Federal University of São Paulo/Paulista School of Medicine, São Paulo, Brazil; 2 Augenärzte Graubünden, Davos, Switzerland; 3 Sierra Eye Associates, University of Nevada, Reno School of Medicine, Reno, NV USA

**Keywords:** Avacincaptad pegol, Glaucoma, Intraocular pressure, Pegcetacoplan, Retinal nerve fiber layer

## Abstract

The approval of Syfovre® (pegcetacoplan) and Iverzay® (avacincaptad pegol) for the treatment of geographic atrophy (GA) marks a significant advancement in retinal disease therapy, offering both complement 3 and complement 5 inhibitors. With this breakthrough, an increase in intravitreal injections (IVI) is expected to treat GA, raising questions about potential effects on intraocular pressure (IOP). This concern is exacerbated by the larger injection volume required for GA treatment, potentially impacting IOP. Previous studies have shown that IVI can lead to a temporary increase in IOP with a 0.05 ml injection. This transient elevation is challenging to manage with glaucoma drops, and a preventive approach, such as paracentesis immediately before IVIs, may be more effective. Despite concerns, clinical significance and long-term effects of IOP changes with a 0.05 ml injection remain uncertain. To address these concerns, routine evaluations including macular optical coherence tomography (OCT), fundus autofluorescence, IOP measurements, and retinal nerve fiber layer OCT before the first IVI with avacincaptad pegol and pegcetacoplan are recommended to detect potential changes early. Further research is needed to determine the extent to which IOP changes impact GA patients and whether cumulative effects occur with repeated IVIs, especially in those with additional eye conditions.

## Background

With the approval of Syfovre® (pegcetacoplan) by the U.S. Food and Drug Administration (FDA) in February 2023 and Iverzay® (avacincaptad pegol) in August 2023 for clinical use in patients with geographic atrophy (GA), it is now possible for the first time to treat the disease with either a complement 3 or a complement 5 inhibitor [1, 2].

## Main text

Since GA is a widespread disease, an increasing number of intravitreal injections (IVI) is expected, which must be applied regularly at four to eight-week intervals according to the FDA label. However, the new form of therapy will not only expand our spectrum of treatable retinal diseases and significantly increase the number of injections administered annually, but it will also be administered with twice the volume, which could potentially be associated with a higher increase in intraocular pressure (IOP). In particular, older GA patients with inherently poorer perfusion may repeatedly develop short-term increases in IOP with additional retinal nerve fiber layer (RNFL) loss and glaucoma development/progression during larger volume injections [3]. While vascular disease is usually administered with anti-VEGF drugs with a volume of 0.05 mL, GA is treated with both drugs with a volume of 0.1 mL. Therefore, there are some questions in the community that we would like to explain and discuss from our first clinical experience with these drugs.

Millions of intravitreal injections are administered annually to patients with various retinal diseases. It is known from previous clinical studies that immediately after IVI of 0.05 ml, IOP can increase to 50 mmHg [4]. This temporary steep peek of IOP can only be inadequately cushioned by the administration of glaucoma drops, as their pharmacological effect starts slow and cannot prevent a rapid sudden increase. The quickest approach to prevent a postoperative ad hoc increase in IOP is to use paracentesis immediately before an IVI [5]. As early as 2018, a report by the American Academy of Ophthalmology evaluated the temporary increased IOP and possible consequences on the optic nerve. However, their clinical significance and long-term effects remained unknown for an applied volume of 0.05 ml [6]. After unilaterally repeated IVIs, a significant decrease in nerve fiber layer thickness after 2 years was documented in a bilateral comparison [7]. A systematic review showed a 2-fold increase in the risk of sustained IOP elevation in eyes undergoing repeated IVI. The authors also found that the longer the follow-up duration, the higher the risk ratio for this elevation [8].

Due to the recent doubling of the injection volume of several novel drugs, there may be a greater increase in IOP. No pathological changes directly attributed to IOP were observed in the pivotal OAKS and DERBY (pegcetacoplan), and GATHER 1 and GATHER 2 (avacincaptad pegol) studies, which monitored a period of up to two years, but optical coherence tomography (OCT) was not performed in these trials to monitor the RNFL [1, 2, 9]. However, experimental studies with similar higher volume showed a consecutively higher increase in IOP [10–13]. For example, Knip and Välimäki reported an increase in IOP from an average of 14.6 mmHg (SD, 2.4) to 47.1 mmHg (SD, 24.1) without paracentesis just 2 min after IVI of 0.09 ml pegaptanib (Macugen, Pfizer) [10]. Kotliar et al. also reported an increase in IOP of 40.6 mmHg immediately after IVI of 0.1 ml (SD, 12.1) and a reduction to 9.4 mmHg (SD, 4.9) after paracentesis. They also showed in a biomechanical model the exponential increase in IOP values with the reduced length of the globe [11]. Lorenz et al. required an additional paracentesis in 78 (33%) out of 234 consecutive cases after 0.1 ml bevacizumab IVIs to reduce the IOP [12]. Another recent publication showed an estimated IOP rise ranging from 32.3 (SD, 1.4) mmHg for 20-µL injection volume to 76.5 (SD, 1.0) mmHg for 80-µL injection volume in an experimental eye model [13]. For a 50-µL volume, the peak pressure was 50.7 (SD, 0.1) mmHg, and the pressure rise lasted for an average of 28 min (SD, 2).

The clinical benefit-risk ratio of paracentesis prior to intravitreal injection has been discussed repeatedly among experts [14]. However, there is a general consensus that eyes with glaucoma, increased IOP or shortened axial length are potentially more likely to develop higher IOP and chronic-cumulative RNFL damage after multiple consecutive IVI and that paracentesis can therefore be useful on an individual basis. Paracentesis is now generally a safe method of normalizing eye pressure in the event of a sharp increase in eye pressure or acute vision loss. Limited research has been done on the extent to which a temporarily moderate increase in IOP leads to subclinical occult changes in the optic nerve, and whether repeated IVIs individually lead to cumulative effects therefore requires systematic observation of all GA patients with OCT. Physicians should also be aware that patients with neovascular age-related macular degeneration (AMD) might have a reduced RNFL without any IVI [15]. Since some patients with GA may also present with neovascular AMD, some RNFL loss over time may also be found and should not be misconceived.

Finally, we would like to point out that a few cases of ischemic optic neuropathy were found in the pivotal trials OAKS and DERBY with pegcetacoplan [1, 16]. Besides some unanticipated drug-related predisposing factor, a reduced ocular blood flow secondary to an increased IOP should be regarded as a plausible cause. In agreement with this hypothesis, Chen et al. (2019) have shown that a greater number of IVI of antiangiogenic agents leads to increased likelihood of developing ischemic optic neuropathy, and they also attributed it to a higher IOP [17].

## Conclusions

We recommend that in addition to macular OCT, fundus autofluorescence (FAF), IOP measurement and RNFL OCT be routinely carried out before the first IVI with either avacincaptad pegol or pegcetacoplan is performed in order to detect possible changes at an early stage after repeated injections. It remains to be seen to what extent these experimental results will play a role in all GA patients or only individual patients with additional diseases of the eye.

## Data Availability

Not applicable.

## Abbreviations

AMD: Age-related macular degeneration
FA: Fundus autofluorescence
FDA: Food and Drug Administration
GA: Geographic atrophy
IVI: Intravitreal injection
IOP: Intraocular pressure
OCT: Optical coherence tomography
